# 

**Published:** 1904-08

**Authors:** 


ESTABLISHED IN 1839

THE OLDEST DENTAL MAGAZINE IN THE WORLD

INDEPENDENT---------PUBLISHED BY DENTISTS FOR DENTISTS

VOLUME XXXV

NUMBER 8

THIRD SERIES

AUGUST

1904

EDITOR:
WM. GIRD BEECROFT, D. D. S., Madison, Wis.
ASSOCIATE EDITORS:
ALBERT H. KETCHAM, D. D. S., Denver, Colo.
EMORY A. BRYANT, D. D. S., Washington, D. C.
GEO. S. MARTIN, D. D. S., L. D. S., Toronto Junction, Ontario.
Published on the 10th day of each month.
Subscription Price, $1.00 per year.
Foreign Countries, $1.50 per year.
Address all communications, both business and editorial to the editor.
**Entered as Second Class Matter, Madison, Wis.**

